# Tools to Predict Unilateral Primary Aldosteronism and Optimise Patient Selection for Adrenal Vein Sampling: A Systematic Review

**DOI:** 10.1111/cen.15225

**Published:** 2025-03-18

**Authors:** Elisabeth Ng, Stella May Gwini, Winston Zheng, Peter J. Fuller, Jun Yang

**Affiliations:** ^1^ Centre for Endocrinology & Metabolism Hudson Institute of Medical Research Clayton Australia; ^2^ Department of Endocrinology Monash Health Clayton Australia; ^3^ Department of Molecular and Translational Science Monash University Clayton Australia; ^4^ School of Public Health and Preventive Medicine Monash University Melbourne Australia; ^5^ Department of Medicine Monash University Clayton Australia

**Keywords:** adrenal cortex function tests, adrenalectomy, clinical decision‐making, diagnosis, hyperaldosteronism, systematic review

## Abstract

**Objective:**

Primary aldosteronism (PA), the most common endocrine cause of hypertension, is evaluated using adrenal vein sampling (AVS), to determine if aldosterone excess is bilateral or unilateral. AVS is invasive and technically challenging; it would ideally be used only in those with unilateral PA who are candidates for surgical cure. Those with bilateral PA would benefit from a direct path to medical management before AVS. Strategic patient selection for AVS would enable judicious and cost‐efficient use of this procedure. This review evaluates the diagnostic accuracy of published algorithms that aim to predict unilateral PA and therefore facilitate informed selection for AVS.

**Design:**

This systematic review was performed by searching Medline and EMBASE databases to identify published models that sought to subtype PA (PROSPERO registration CRD42021277841). Algorithms reported to predict unilateral PA and therefore select patients for AVS, using AVS and/or surgical outcomes as the gold standard, were systematically evaluated.

**Results:**

There were 28 studies evaluating 63 unique predictive algorithms, of which 14 were tested in multiple cohorts. These were grouped into 5 categories; those combining biochemical, radiological and demographic characteristics, those involving confirmatory testing those using biochemical results only, those involving dynamic testing, and anatomical imaging. The algorithm with the highest sensitivity for unilateral PA which has been validated in at least two cohorts, involved serum potassium, CT imaging, PAC, ARR and female sex (sensitivity 78‐96%). In a hypothetical scenario of 1000 people with PA where 30% have unilateral PA, this top performing algorithm would appropriately select 234−289 people for AVS and allow 143−324 to correctly bypass AVS.

**Conclusions:**

Accurate algorithms to inform selection for AVS will ensure that AVS is only performed in patients with a high probability of unilateral PA without clear evidence of the side of lateralisation. This will lower the demand for this invasive procedure, avoid unnecessary procedural complications, and reduce associated health care costs. Further validation of the top‐performing algorithms in larger and diverse cohorts will support their use in routine practice.

**Abbreviations**
ACTH: adrenocorticotropic hormone
ARR: aldosterone‐to‐renin ratio
AVS: adrenal vein sampling
CCT: captopril challenge test
CT: computed tomography
DRC: direct renin concentration
eGFR: estimated glomerular filtration rate
LI: lateralisation index
MR: mineralocorticoid receptor
MRI: magnetic resonance imaging
NPV: negative predictive value
PA: primary aldosteronism
PAC: plasma aldosterone concentration
PET: positron emission tomography
PPV: positive predictive value
PRA: plasma renin activity
PRISMA: Preferred Reporting Items for Systematic Reviews and Meta‐Analysis
RF: random forest
SST: saline suppression test
SUVmax: maximum standardised uptake value

## 1 Introduction

Primary aldosteronism (PA), the most common endocrine cause of hypertension, currently involves a diagnostic process that can be complex. This involves a screening blood test, confirmatory testing in some scenarios, and subtyping to determine the laterality of aldosterone excess, with the latter typically achieved with CT imaging and adrenal vein sampling (AVS) [1]. Subtyping of PA is important as lateralised disease can be treated with adrenalectomy while bilateral disease requires long‐term treatment with mineralocorticoid receptor antagonists (MRA). Current official guidance invites all individuals who would be suitable for and accepting of surgical treatment to undergo AVS to facilitate possibly curative treatment (laparoscopic adrenalectomy in most cases) [1]. Multiple algorithms have been described to predict unilateral PA, and some can be applied to improve selection for AVS. Strategic patient selection for AVS would enable the judicious and cost‐efficient use of this procedure which is resource‐intensive, invasive, and limited in availability [2].

This systematic review examines the literature for algorithms which have been evaluated for their accuracy in predicting unilateral PA. To identify all patients who can benefit from AVS and potential surgical cure, the preferred algorithm should have high sensitivity for unilateral PA.

## 2 Methods

### 2.1 Search Strategy and Selection Criteria

**Figure 1:**
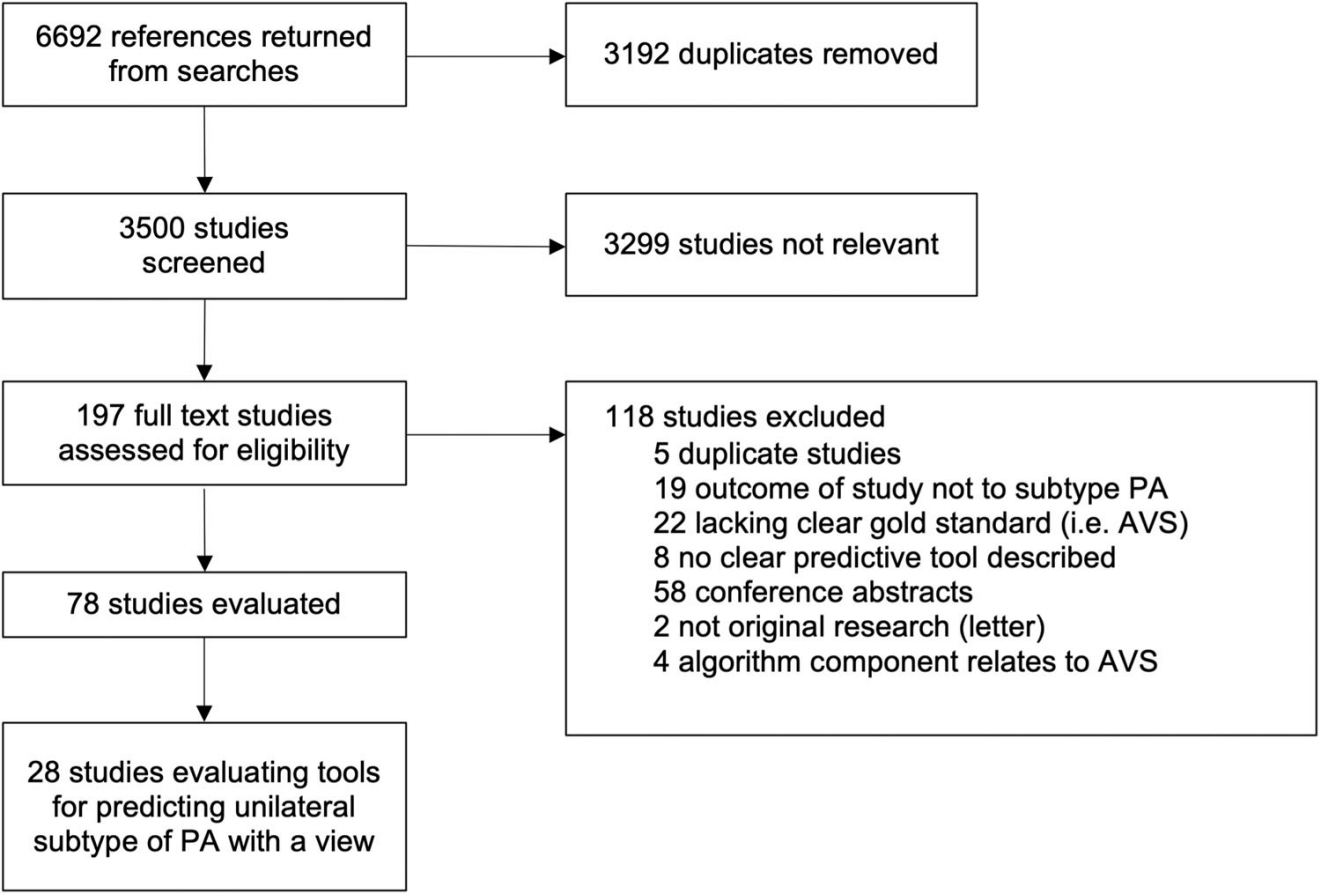
PRISMA flow chart.

This systematic review was performed and reported in accordance with Preferred Reporting Items for Systematic Reviews and Meta‐Analysis (PRISMA) guidelines [3]. PROSPERO registration was completed before project commencement (CRD42021277841). Medline and EMBASE were searched, from inception to 6 October 2023. The search methodology has been previously published [4] and search terms can be found in Supporting Information S1: Table 1. Two independent investigators screened all studies meeting the inclusion criteria and reviewed full text articles. Data extraction was performed for all studies by one investigator (E.N.), with a second investigator (W.Z.) independently reviewing 20% of the studies. Discrepancies at each step were resolved by a third independent investigator (J.Y.). This systematic review includes all studies evaluating a predictive algorithm for unilateral PA, where the application would be to inform selection for AVS rather than to bypass AVS (Figure 1).

### 2.2 Data Extraction

As previously described [4], data extracted included study details (authors, country, study type), participant clinical and demographic data, PA work up approach (screening and confirmatory testing methods), pathology results (aldosterone, renin, aldosterone‐to‐renin ratio [ARR], potassium, creatinine, estimated glomerular filtration rate [eGFR]), CT imaging, AVS and histopathology, details of the predictive tool used, figures used to calculate sensitivity, specificity, positive predictive value (PPV) and negative predictive value (NPV) where available and author conclusions. In the reporting of PAC and ARR, conversion of plasma aldosterone concentration (PAC) from ng/dL to pmol/L utilised a conversion factor of 27.74, and conversion from plasma renin activity (PRA) as ng/mL/h to direct renin concentration (DRC) as mU/L utilised a factor of 8.2 [1].

### 2.3 Data Analysis

Predictive tools were grouped into five classes based on the parameters used to define the algorithms; that is, tools combining biochemical, radiological and demographic characteristics, those involving confirmatory testing, those using blood or urine test results only, those involving ACTH or postural stimulation testing and those involving anatomical imaging.

Algorithms developed and then validated in at least two additional cohorts were assessed using forest plots and the number of cases correctly or unhelpfully sent for AVS were calculated in a hypothetical group of 1000 patients with PA. Published maximum and minimum sensitivities and accompanying specificities were applied to the cohort, assuming a 30% prevalence of unilateral disease.

**Figure 2:**
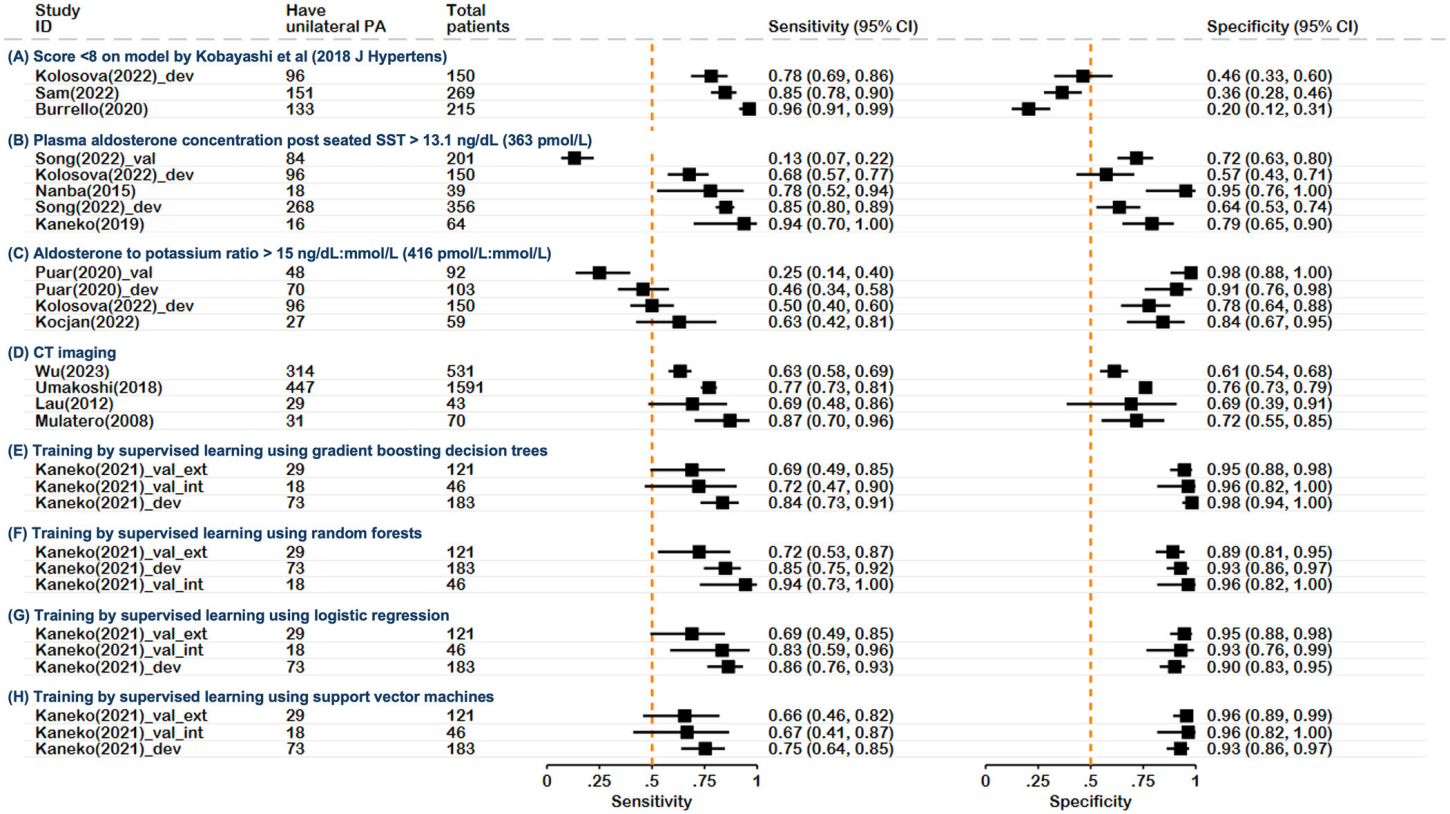
Forest plot of algorithms that have been assessed in three or more cohorts. CI, confidence interval; CT, computed tomography; dev, development cohort; ext: external cohort; int, internal cohort; PA, primary aldosteronism; SST, saline suppression test; Val, validation cohort.

**Figure 3:**
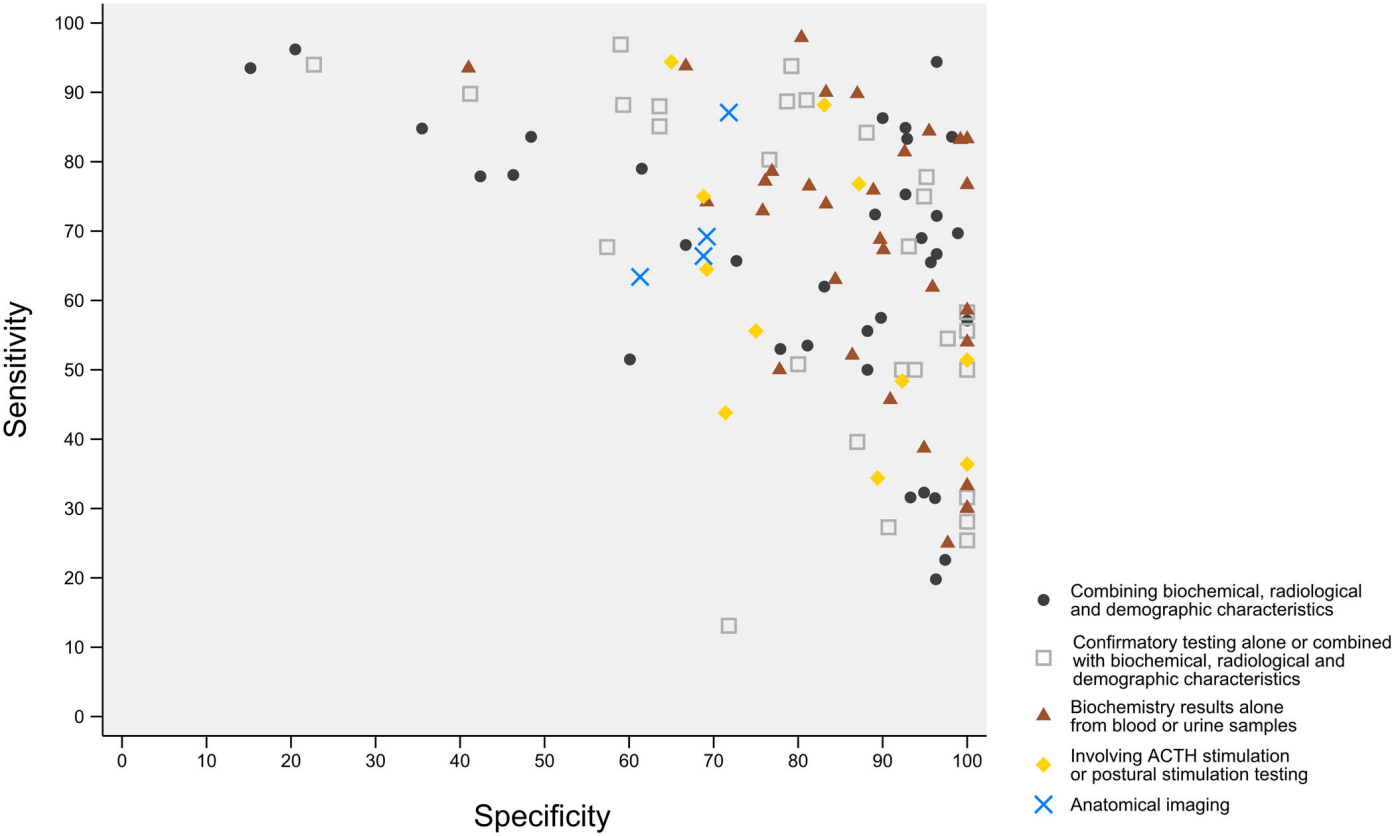
Scatterplot of sensitivity vs specificity for all algorithms.

Risk of bias was assessed using the Quality Assessment of Diagnostic Accuracy Studies‐2 tool [5]. The predictive tool was considered the index test and the gold standard test considered the reference standard. Where data collected during the work‐up of PA was used in the index test, an appropriate interval between the index test and reference standard (AVS) was assumed. Risk of bias and applicability concerns were stated to be unclear where relevant information was not provided.

### 2.4 Role of the Funding Source

Funders had no role in study design, data collection, data analysis, data interpretation or manuscript writing.

## 3 Results

There were 28 studies included in this review (Figure 1), evaluating 63 unique predictive algorithms/tools, of which 14 were tested in multiple cohorts, 11 tested in multiple studies and 14 had variations in criteria, for example, different cut‐offs using the same scoring system or different methods of analysis using the same form of imaging. The 63 unique tools were derived from 111 tools in total, differing by either cohort (training and validation), algorithm parameters or variation in scores applied to the same algorithm (Supporting Information S1: Table 2). There were 31 unique patient cohorts from the 28 studies. The sensitivity and specificity of tools which have been tested in three or more cohorts are represented in Figure 2, while the relative sensitivity and specificity of different categories of all 63 tools, as described below, are shown in a scatterplot (Figure 3).

### 3.1 Study Characteristics

The 28 studies were published between 2008 and 2023. Most were single‐centre studies (20/28) from referral centres in Japan (12/28), Europe (9/28) and China (4/28). All studies were cohort or cross‐sectional studies, and the majority were conducted retrospectively (20/28). All studies relied on AVS for subtype determination, with 6/28 studies also using surgical outcome data in addition to AVS; three of these applied the PASO consensus and three were conducted before publication of the PASO guidelines; they assessed histopathology and/or post‐operative aldosterone, renin, potassium, and/or blood pressure.

Screening for PA involved either an ARR (15/28) alone or combined with a minimum PAC (7/28), while confirmation of PA was mostly by saline suppression testing (SST) (23/28). All algorithms which achieved a sensitivity of 90% or greater are detailed in Table 1; sensitivity ≥ 90% was reported with 11 predictive tools from 10 studies. Only the model by Nanba et al. using a score ≥ 3 had a sensitivity over 90% in more than one cohort [9].

**Table 1:** Algorithms with a sensitivity over 90% for predicting unilateral primary aldosteronism.

Algorithm	Author(year) and cohort if more than one	Sensitivity	Specificity
Score of < 8 on Model A by Kobayashi et al. [6] tested by Burrello et al. [7] 1. Serum potassium > 3.9 mmol/L = 4‐points 2. Serum potassium 3.5−3.9 mmol/L = 3‐points 3. Baseline PAC < 210 pg/mL (583 pmol/L) = 2‐points 4. Baseline ARR (PAC/PRA) < 620 pg/mL:ng/mL/h = 2‐points 5. Female sex = 1‐point	Burrello (2020)	0.962	0.205
Hypertension duration ≥ 8 years and no apparent adrenal tumour on CT [8]	Okamoto (2021)	0.935	0.152
Score ≥ 3 on a model by Nanba et al. [9] and validated by Song et al. [10] 1. Serum potassium ≤ 3.4 mEq/L = 2‐points 2. PAC ≥ 165 pg/mL (458 pmol/L) = 3‐points 3. ARR post‐CCT ≥ 1000 pg/mL:ng/mL/h (2774 pmol/L:ng/mL/h) = 3‐points	Nanba (2014)	0.969	0.590
	Song (2022)	0.940	0.227
PAC post‐SST > 13.1 ng/dL (363 pmol/L) [11]	Kaneko (2019)	0.938	0.792
PAC 2 h post‐SST > 61 pg/mL (169 pmol/L) [12]	Nanba (2015)	1.000	NA
PAC 4 h post‐SST > 80 pg/mL (222 pmol/L) [12]	Nanba (2015)	1.000	NA
PAC > 25 ng/dL (694 pmol/L) and urinary aldosterone > 30 ug/24h [13]	Mulatero (2008)	0.935	0.410
PAC ≥ 166 pg/mL (460 pmol/L) [9]	Nanba (2014)	0.938	0.667
Supervised learning using a random forest model to evaluate clinical data and peripheral blood biomarkers [14]	Kaneko (2021) ‐ internal validation cohort	0.944	0.964
Random forest classification trees using peripheral plasma steroid concentrations [15]	Yang (2019)	0.979	0.804
PAC > 605.7 pg/mL (1680 pmol/L) after ACTH stimulation [16]	Moriya (2017)	0.944	0.650

Abbreviations: ACTH, adrenocorticotrophic hormone; ARR, aldosterone‐to‐renin ratio; CCT, captopril challenge test; CT, computed tomography; PAC, plasma aldosterone concentration; PRA, plasma renin activity; SST, saline suppression test.

### 3.2 Models Combining Biochemical, Radiological and Demographic Characteristics

There were 10 studies reporting on 21 unique algorithms tested in 12 unique cohorts in this category (Supporting Information S1: Table 2). Sensitivity ranged from 19.8% to 96.2%; 7 had been validated in a second cohort, 6 of these in a third cohort. The algorithm with the highest sensitivity of 78−96% across 3 studies was the one by Kobayashi et al. applying a score of < 8 to select for unilateral PA (4‐points for serum potassium > 3.9 mEq/L and 3‐points for 3.5−3.9 mEq/L; 3‐points for no adrenal nodule on CT; 2‐points for screening PAC < 21 ng/dL (583 pmol/L); 2‐points for screening ARR (PAC:PRA) < 62 ng/dL:ng/mL/h; 1‐point for female sex) [7, 17, 37].

### 3.3 Models Involving Confirmatory Testing Alone or Combined With Biochemical, Radiological and Demographic Characteristics

There were 12 studies reporting on 14 unique predictive algorithms tested in 12 unique cohorts in this category. The confirmatory tests used were SST (five studies), captopril challenge test (CCT) (two studies), CCT or SST (three studies), frusemide upright test (one study) and multiple confirmatory tests (one study).

Only two algorithms had been evaluated in multiple cohorts; (1) a score involving serum potassium, PAC and ARR post‐CCT by Nanba et al. [9] and (2) elevated PAC post‐SST as proposed by Kaneko et al. and Nanba et al. [11, 12] A score of ≥ 3 using the model by Nanba et al. (2‐points for serum potassium ≤ 3.4 mEq/L, 3‐points for PAC ≥ 165 pg/mL (458 pmol/L), 3‐points for ARR post‐CCT ≥ 1000 pg/mL:ng/mL/h (2774 pmol/L:ng/mL/h)) had a sensitivity of 97% when originally evaluated in a Japanese population [9] and 94% when evaluated in a Chinese cohort by Song et al. (Figure 2) [10]. PAC post‐SST has been proposed as a method to select the unilateral subtype of PA with similar cut‐offs suggested Nanba et al. (> 132 pg/mL [366 pmol/L]) and Kaneko et al. (> 13.1 ng/mL [363 pmol/L]) [11, 12]. In the development cohorts, Nanba et al. and Kaneko et al. reported sensitivities of 77.8% and 93.8% (and specificities of 95.2% and 79.2%), respectively [11, 12]. When tested in three external cohorts, Song et al. reported a sensitivity of 85.1% in a Chinese cohort but only 13.1% in an Australian validation cohort [10], while Kolosova et al. reported a sensitivity of 67.7% and specificity of 57.4% for this same criterion in a Czech cohort [17].

### 3.4 Biochemical Results Alone From Blood or Urine Samples

Algorithms to predict unilateral PA based on blood or urine results alone were reported in 12 studies, with 16 unique algorithms studied in 13 cohorts. The algorithms with a sensitivity ≥ 90% were 6 am PAC > 217.5 pg/mL (603 pmol/L) (sensitivity 90.0%, specificity 83.3%) [18], PAC > 25 ng/dL (694 pmol/L) and urinary aldosterone > 30 ug/day (sensitivity 93.5%, specificity 41.0%) [19], PAC ≥ 166 pg/mL (460 pmol/L) (sensitivity 93.8%, specificity 66.7%) [9] and a prediction model using peripheral venous steroid profiling (sensitivity 97.9%, specificity 80.4%) [15].

Biochemical parameters that had been tested in multiple cohorts were hypokalaemia, a basal PAC above a certain threshold, elevated urinary aldosterone and an elevated aldosterone‐to‐lowest‐potassium ratio. Hypokalaemia was evaluated at thresholds of < 3.0 mEq/L [19], < 3.4 mEq/L [20], < 3.5 mEq/L [9, 13] and ≤ 3.6 mEq/L [19]. Of these studies, the highest diagnostic accuracy was reported in the smallest study (*n* = 35, 18 unilateral), with Minami et al. reporting a sensitivity of 83.3% and specificity of 100% for unilateral PA based on serum potassium < 3.4 mEq/L [20]. The largest study (*n* = 70, 31 unilateral) by Mulatero et al. tested serum potassium < 3.5 mEq/L and reported a sensitivity of 77.2% and specificity of 76.1% [13]. The thresholds of < 3.0 mEq/L and ≤ 3.6 mEq/L were examined in the same cohort, with the latter having a sensitivity of 74.2% and specificity of 69.2% while the lower cut‐off had a sensitivity of 38.7% and specificity of 94.9% [19].

PAC above a specified threshold was evaluated at cut‐offs of 166 pg/mL (460 pmol/L) [9], 18 ng/dL (499 pmol/L) [20], 21.5 ng/dL (596 pmol/L) [21] and 394 pg/mL (1093 pmol/L) [18]. The lowest cut‐off (460 pmol/L) had a sensitivity of 93.8% and specificity of 66.7% [9] while the highest cut‐off had a sensitivity of 76.7% and specificity of 100% [18]. Urinary aldosterone was evaluated at ≥ 9 ug/day [20], > 14.6 ug/day and > 22 ug/day [18] with sensitivities of 78.6%, 75.9% and 58.6%, respectively, and accompanying specificities of 76.9%, 88.9% and 100%. The aldosterone‐potassium ratio described by Puar et al. had sensitivities between 52.1% and 72.9% and specificities 75.8%−86.4% for a cut‐off > 10 ng/dL:mmol/L (277 pmol/L:mmol/L), and a higher cut‐off of > 15 ng/dL:mmol/L (416 pmol/L:mmol/L) had lower sensitivities (25%−45.7%) and higher specificities (90.9%−97.7%) [22]. An aldosterone‐potassium ratio > 15 ng/dL:mmol/L (416 pmol/L:mmol/L) was validated by Kocjan et al. and Kolosova et al. who reported higher sensitivities (50%−63%) and lower specificities (77.8%−84.4%) [17, 23].

### 3.5 Models Involving ACTH Stimulation or Postural Stimulation Testing

There were seven studies that used either the postural stimulation test [19, 24, 25, 26] or ACTH stimulation test to predict unilateral PA (Supporting Information S1: Table 2) [11, 16, 27]. None of the 11 algorithms reported were tested in more than one cohort. A sensitivity > 90% was only reported by Moriya et al. with a maximum PAC > 605.7 pg/mL (1680 pmol/L) after ACTH stimulation (250 ug of synthetic ACTH intravenously with blood tests every 30 min for 2 h) [16].

### 3.6 Anatomical Imaging Alone

Adrenal CT to subtype PA was studied in four studies which specified the purpose to select for AVS rather than bypass AVS, with sensitivities of 63.4%−87.1% and specificities of 61.3%−71.8% [13, 19, 25, 26].

### 3.7 Assessment of Study Quality and Publication Bias

Most of the studies displayed moderate to high risk of bias, as assessed by the QUADAS‐2 score (Supporting Information S1: Figure 1).

### 3.8 Clinical Implications of Algorithms for AVS Selection in a Hypothetical Cohort

To contextualise the diagnostic accuracy of algorithms tested in at least two cohorts, published sensitivities and specificities were applied to a hypothetical cohort of 1000 patients with PA, using the maximum and minimum sensitivity (Table 2). The algorithm with highest sensitivity (96%), by Kobayashi et al. would correctly send 289/300 for AVS and allow 143/700 to correctly bypass AVS, while 11 would have incorrectly bypassed AVS and 557 with bilateral disease would have been referred for AVS. The random forest model by Kaneko et al. had the second highest sensitivity at 94%; this would have allowed 283/300 to be correctly sent for AVS and 675/700 to correctly bypass AVS, with 17 with unilateral disease inaccurately bypassing AVS and 25 with bilateral disease being unhelpfully sent for AVS.

**Table 2:** Applying published maximum and minimum sensitivities of algorithms validated in at least two cohorts in a hypothetical 1000‐person population with primary aldosteronism (30% prevalence of unilateral disease).

Algorithm	Study	Diagnostic accuracy reported by study		Unilateral cases (*N* = 300)		Bilateral cases (*N* = 700)		Number of bilateral cases seen per unilateral found*
		Sensitivity	Specificity	Inaccurately bypassed AVS	Correctly sent for AVS	Unhelpfully sent for AVS	Correctly bypassed AVS	
Score by Kobayashi et al. score of < 8: 1. Female = 1‐point 2. Plasma aldosterone concentration < 210 pg/mL (583 pmol/L) = 2‐points 3. Aldosterone‐to‐renin ratio < 620 pg/mL per ng/mL/h (1720 pmol/L per ng/mL/h) = 2‐points 4. Serum potassium > 3.9 mmol/L = 4‐points 5. Serum potassium 3.5−3.9 mmol/L = 3‐points 6. No adrenal nodules (>1 cm) on CT = 3‐points	Maximum sensitivity ‐ Burrello (2020)	96.2	20.5	11/300 (3.7%)	289/300 (96.3%)	557/700 (79.6%)	143/700 (20.4%)	557/289 (1.9:1)
	Minimum sensitivity ‐ Kolosova (2022)_dev	78.1	46.3	66/300 (22.0%)	234/300 (78.0%)	376/700 (53.7%)	324/700 (46.3%)	376/234 (1.6:1)
Plasma aldosterone concentration post seated saline suppression test > 13.1 ng/dL (363 pmol/L)	Maximum sensitivity ‐ Kaneko (2019)	93.8	79.2	19/300 (6.3%)	281/300 (93.7%)	146/700 (20.9%)	554/700 (79.1%)	146/281 (0.5:1)
	Minimum sensitivity ‐ Song (2022)_val	13.1	71.8	261/300 (87.0%)	39/300 (13.0%)	197/700 (28.1%)	503/700 (71.9%)	197/39 (5.1:1)
Aldosterone to potassium ratio > 15 ng/dL:mmol/L (416 pmol/L:mmol/L) (baseline plasma aldosterone concentration to lowest potassium ratio)	Maximum sensitivity ‐ Kocjan (2022)	63.0	84.4	111/300 (37.0%)	189/300 (63.0%)	109/700 (15.6%)	591/700 (84.4%)	109/189 (0.6:1)
	Minimum sensitivity ‐ Puar (2020)_val	25.0	97.7	225/300 (75.0%)	75/300 (25.0%)	16/700 (2.3%)	684/700 (97.7%)	16/75 (0.2:1)
Unilateral lesion on CT	Maximum sensitivity ‐ Mulatero (2008)	87.1	71.8	39/300 (13.0%)	261/300 (87.0%)	197/700 (28.1%)	503/700 (71.9%)	197/261 (0.8:1)
	Minimum sensitivity ‐ Wu (2023)	63.4	61.3	110/300 (36.7%)	190/300 (63.3%)	271/700 (38.7%)	429/700 (61.3%)	271/190 (1.4:1)
Training by supervised learning: Gradient boosting decision trees	Maximum sensitivity ‐ Kaneko (2021)_dev	83.6	98.2	49/300 (16.3%)	251/300 (83.7%)	13/700 (1.9%)	687/700 (98.1%)	13/251 (0.1:1)
	Minimum sensitivity ‐ Kaneko (2021)_val_ext	69.0	94.6	93/300 (31.0%)	207/300 (69.0%)	38/700 (5.4%)	662/700 (94.6%)	38/207 (0.2:1)
Training by supervised learning: Random Forest model	Maximum sensitivity ‐ Kaneko (2021)_val_int	94.4	96.4	17/300 (5.7%)	283/300 (94.3%)	25/700 (3.6%)	675/700 (96.4%)	25/283 (0.1:1)
	Minimum sensitivity ‐ Kaneko (2021)_val_ext	72.4	89.1	83/300 (27.7%)	217/300 (72.3%)	76/700 (10.9%)	624/700 (89.1%)	76/217 (0.4:1)
Training by supervised learning: Logistic regression	Maximum sensitivity ‐ Kaneko (2021)_dev	86.3	90.0	41/300 (13.7%)	259/300 (86.3%)	70/700 (10.0%)	630/700 (90.0%)	70/259 (0.3:1)
	Minimum sensitivity ‐ Kaneko (2021)_val_ext	69.0	94.6	93/300 (31.0%)	207/300 (69.0%)	38/700 (5.4%)	662/700 (94.6%)	38/207 (0.2:1)
Training by supervised learning: Support vector machines	Maximum sensitivity ‐ Kaneko (2021)_dev	75.3	92.7	74/300 (24.7%)	226/300 (75.3%)	51/700 (7.3%)	649/700 (92.7%)	51/226 (0.2:1)
	Minimum sensitivity ‐ Kaneko (2021)_val_ext	65.5	95.7	103/300 (34.3%)	197/300 (65.7%)	30/700 (4.3%)	670/700 (95.7%)	30/197 (0.2:1)

Abbreviations: AVS, adrenal vein sampling; CT, computed tomography; dev, development cohort; ext, external cohort; val, validation cohort.

*Number of bilateral cases sent for AVS compared to unilateral cases identified by AVS, if patients were selected for AVS using the algorithm being tested. In this hypothetical scenario, if all patients were subjected to AVS, the number of bilateral cases seen per unilateral found would be 700:300 (2.3:1).

## 4 Discussion

This systematic review examines published algorithms for predicting unilateral PA to identify patients who would benefit most from AVS. There were 28 studies included, with prioritisation of high sensitivity over specificity so as to avoid missing any patients with unilateral PA.

The top performing algorithms incorporated sex, PAC, ARR, serum potassium, CT imaging, PAC post‐SST and ARR post‐CCT [7, 9, 11]. The use of baseline data only in certain algorithms, for example the algorithm by Kobayashi et al. [6] allows prediction of PA subtype without confirmatory testing. This would offer an advantage in many clinical settings as it would save eligible patients a time‐consuming test and expedite their referral for AVS. Key elements of the algorithm by Kobayashi et al, including serum potassium, PAC and ARR, were also identified in a study using machine learning as the strongest predictors of unilateral PA [28].

The model by Nanba et al. was evaluated in three studies and a score of ≥ 3 had high sensitivity (94%−97%) in two studies [9, 10]; the third study assessed different scores and was not directly comparable [17]. This algorithm shared PAC and serum potassium as mutual factors with the algorithm by Kobayashi et al. [6, 17] but the latter did not include confirmatory test results. Patients with a score < 8 using the model by Kobayashi et al. based on initial screening results could proceed to AVS while others who proceed to confirmatory testing could have their likelihood of unilateral PA re‐assessed using the algorithm by Nanba et al. The effectiveness of such an approach remains to be tested but it may help to reduce demand for both confirmatory testing and AVS.

In this review, we prioritised algorithms with the highest sensitivity so that patients with likely unilateral PA do not miss out on AVS. In our 1000‐persons scenario, the model by Kobayashi et al. would permit 20%−46% fewer unhelpful AVS procedures (i.e., AVS correctly bypassed) while 78%−96% of unilateral disease will be discovered, with less than two cases of bilateral disease found for every case of unilateral disease identified by AVS (Table 2). This algorithm may be highly desirable to a well‐resourced centre, based on its ability to detect most patients with PA, with 4%−22% of unilateral cases being missed. However, in low resource settings where AVS availability is highly restricted, algorithms with high specificity may be preferred for limiting AVS to those with a very high probability of unilateral PA. In our hypothetical scenario, the aldosterone‐to‐potassium ratio would allow 85%−98% of patients with bilateral PA to bypass AVS, at the expense of missing 37%−75% of those with unilateral PA. This algorithm would maximise the likelihood that each AVS performed would reveal a unilateral source of PA, with only 0.2−0.6 bilateral cases for every unilateral case identified by AVS (i.e., 1.7−4.7 unilateral cases detected for every bilateral case sent for AVS) (Table 2). Indeed, all of these algorithms simply present a probability—the higher the aldosterone concentration and ARR (either at baseline or following suppression testing), and the lower the potassium concentration, the more likely it is for a patient to have unilateral PA. Machine learning is becoming increasingly useful as a tool for identifying factors highly correlated to unilateral PA, and therefore allowing creation of models that encompass a variety of patient factors [14, 29]. Advances in technology will contribute to development of clinically accessible tools that enable accurate triage of patients towards optimal management of their PA, either with AVS for subtyping, upfront medical management or upfront surgical referral.

Before machine learning becomes widely available, the use of a simple algorithm to inform patient selection for AVS will help reduce the impact of the bottleneck posed by AVS, being an invasive and resource‐intensive procedure that can be limited in availability [30]. The algorithms can aid patient counselling and clinician decision‐making so that only patients with likely unilateral PA are referred for AVS. A high probability of unilateral PA based on the algorithms, in the absence of a visible adrenal lesion on imaging, may even prompt referral to another centre where AVS is available, if a surgical cure is desired. The choice of subtyping modality will also depend on patient preference after being fully informed about the diagnostic accuracy of a non‐invasive method of subtype prediction as compared to AVS or other alternatives. On the horizon are novel methods to subtype PA such as nuclear imaging [31, 32, 33], miRNA [34] and proteomics [35] (and multiomics), but none are yet available for routine clinical use.

A limitation of this study is the heterogeneity in the included studies, particularly in methodologies for diagnosing and subtyping PA, and measuring analytes in blood or urine. Due to this variability, we did not perform a meta‐analysis to estimate aggregated diagnostic accuracy. Most studies were retrospective and conducted in referral centres, introducing selection bias. Use of AVS as a comparator in all studies inherently excludes patients with PA who were not subtyped, thereby omitting their characteristics from algorithm development or validation. Additionally, AVS has its own limitations, as it may not always identify unilateral disease curable by adrenalectomy [32, 36]. Although the PASO criteria provide a framework for assessing post‐surgical response, only three of 28 studies applied them and another three used biochemical and/or clinical post‐operative criteria for subtype confirmation. Most algorithms originated from single centre studies, with few validated across multiple cohorts or diverse populations, limiting their generalisability. We acknowledge that variation in centre‐specific population characteristics and protocols are both a real‐life issue and a study limitation. For this reason we have focused on algorithms validated in at least two cohorts, with their data presented in a forest plot (Figure 2) and their utility demonstrated in a 1000‐person hypothetical scenario (Table 2).

Future work to develop and validate algorithms that estimate the probability of unilateral vs bilateral PA will better inform the need for AVS. For now, our analysis of published algorithms to inform selection for AVS can help reduce the demand for this challenging procedure, minimise unnecessary procedural complications, and reduce associated health care costs. It may also improve equity of access to AVS and support patients in making informed decisions about undergoing, or bypassing, this invasive procedure. Existing algorithms require further validation in large and more diverse cohorts before being routinely implemented in practice.

## Conflicts of Interest

The authors declare no conflicts of interest.

## Supporting information

Supporting information.

## Data Availability

The data supporting this review are available within the main text or supplemental materials, and further details may be sourced from the original articles cited.
