# Supplementary material for: Tools to Predict Unilateral Primary Aldosteronism and Optimise Patient Selection for Adrenal Vein Sampling: A Systematic Review
Source: Clin Endocrinol (Oxf). 2025 Mar 18;103(1):3–12. doi: 10.1111/cen.15225 (PMC12134443; doi:10.1111/cen.15225)

Supporting information

Table S1: Database search terms

Database	Medline (Ovid)		Embase (Ovid)	
Defined headings	"Primary aldosteronism"	"Outcome"	"Primary aldosteronism"	"Outcome"
Search terms	Terms combined with OR:<br><br>Hyperaldosteronism/<br>Hyperaldosteronism<br>Aldosteronism<br>Hyperaldosteronaemia<br>Hyperaldosteronemia<br>Hyper-aldosteronaemia<br>Hyper-aldosteronemia<br>Hyper-aldosteronism<br>Aldosteronaemia<br>Aldosteronemia<br>Conn's syndrome<br>Conn Syndrome<br>Conn* syndrome<br>Conn's disease<br>Conn disease<br>Conn* disease	Terms combined with OR:<br><br>Unilateral<br>Bilateral<br>Lateralis*<br>Lateraliz*<br>Localis*<br>Localiz*<br>Subtyp*	Terms combined with OR:<br><br>Primary Aldosteronism/<br>Hyperaldosteronism/<br>Hyperaldosteronism<br>Aldosteronism<br>Hyperaldosteronaemia<br>Hyperaldosteronemia<br>Hyper-aldosteronaemia<br>Hyper-aldosteronemia<br>Hyper-aldosteronism<br>Aldosteronaemia<br>Aldosteronemia<br>Conn* syndrome<br>Conn* disease	Terms combined with OR:<br><br>Unilateral<br>Bilateral<br>Lateralis*<br>Lateraliz*<br>Localis*<br>Localiz*<br>Subtyp*
Search run	[Primary aldosteronism] AND [Outcome]		[Primary aldosteronism] AND [Outcome]	
Limits	English only		English only	

*truncation wildcard used to search all words with the same preceding letters

Table S2: Algorithms included in systematic review and reported diagnostic accuracy*

Study	Algorithm	Number of patients score was applied to		Diagnostic accuracy			
		Unilateral PA	Bilateral PA	Sensitivity	Specificity	PPV	NPV
**Algorithms combining biochemical, radiological and demographic characteristics**							
Burrello(2020)	Score by Kobayashi et al < 8<br>1) Serum potassium: > 3.9 mEq/L (4 points), 3.5-3.9 (3 points)<br>2) No adrenal nodules on CT (3 points)<br>3) PAC at screening < 21 ng/dL (583 pmol/L) (2 points)<br>4) ARR (PAC:PRA) at screening < 62 ng/dL:ng/mL/h (2 points)<br>5) Female sex (1 point)	133	82	0.962	0.205	0.660	0.773
Kaneko(2021)	Training by supervised learning: Gradient boosting decision trees (GBDT) (training cohort)	73	110	0.836	0.982	0.968	0.900
	Training by supervised learning: Random forests (RF) (training cohort)	73	110	0.849	0.927	0.886	0.903
	Training by supervised learning: Logistic regression (LR) (training cohort)	73	110	0.863	0.900	0.851	0.908
	Training by supervised learning: Support vector machines (SVM) (training cohort)	73	110	0.753	0.927	0.873	0.850
	Training by supervised learning: GBDT (internal validation cohort)	18	28	0.722	0.964	0.929	0.844
	Training by supervised learning: RF (internal validation cohort)	18	28	0.944	0.964	0.944	0.964
	Training by supervised learning: LR (internal validation cohort)	18	28	0.833	0.929	0.882	0.897
	Training by supervised learning: SVM (internal validation cohort)	18	28	0.667	0.964	0.923	0.818
	Training by supervised learning: LR (external validation cohort)	29	92	0.690	0.946	0.800	0.906
	Training by supervised learning: SVM (external validation cohort)	29	92	0.655	0.957	0.826	0.898
	Training by supervised learning: GBDT (external validation cohort)	29	92	0.690	0.946	0.800	0.906
	RF model using 3 variables – serum potassium, PAC, and serum sodium (external validation cohort)	29	92	0.724	0.891	0.677	0.911
Minami(2008)	Age < 52 years AND Serum potassium < 3.4 mEq/L	18	17	0.571	1.000	1.000	0.600
	Age < 52 years	18	17	0.556	0.882	0.833	0.652
	SBP ≥ 148mmHg	18	17	0.500	0.882	0.818	0.625
Mulatero(2008)	1) Hypokalemia (<3 mEq/L) AND<br>2) PAC > 25 ng/dL (694 pmol/L) AND/OR<br>3) Urinary aldosterone > 30 ug/24 h	31	39	0.323	0.949	0.833	0.638

	1) Hypertension of grade 3 or greater AND<br>2) Hypokalemia (<3 mEq/L) AND<br>3) PAC > 25 ng/dL (694 pmol/L) AND/OR<br>4) Urinary aldosterone > 30 ug/24 h	31	39	0.226	0.974	0.875	0.613
Okamoto(2021)	All of:<br>1) Male<br>2) Hypokalaemia (≤ 3.5 mEq/L or taking a potassium supplement)<br>3) ARR ≥ 560 pg/mL:ng/mL/h<br>4) No adrenal tumour	200	1386	0.315	0.962	0.543	0.907
	Age ≤ 55 years AND no apparent adrenal tumour on CT	200	1386	0.515	0.601	0.157	0.896
	Hypertension duration ≥ 8 years AND no apparent adrenal tumour on CT	200	1386	0.935	0.152	0.137	0.942
	Hypokalaemia (serum potassium ≤ 3.5 mEq/L or use of potassium supplementation at diagnosis of PA) AND no adrenal tumour on CT	200	1386	0.620	0.831	0.346	0.938
	Serum potassium ≤ 3.7 mEq/L AND no apparent adrenal tumour on CT	200	1386	0.790	0.615	0.228	0.953
	ARR ≥ 560 pg/mL:ng/mL/h AND no apparent adrenal tumour on CT	200	1386	0.680	0.667	0.227	0.935
	PAC > 223 pg/mL AND no apparent adrenal tumour on CT	200	1386	0.530	0.779	0.257	0.920
Puar(2020)	Algorithm by Kobayashi et al<br>Model A score of ≤ 1 point:<br>1) Serum potassium: > 3.9 mmol/L (4 points); 3.5–3.9 mmol/L (3 points)<br>3) PAC < 210 pg/mL (583 pmol/L) (2 points)<br>4) ARR (PAC/PRA) < 620 pg/mL:ng/mL/h (1720 pmol/L:ng/mL/h) (2 points)<br>5) Female sex (1 point)	70	33	0.657	0.727	0.836	0.500
	Algorithm by Umakoshi et al<br>A) Unilateral disease on CT<br>B) Serum potassium < 3.5 mEq/L	67	31	0.836	0.484	0.778	0.577
Sam(2022)	Algorithm by Mulatero et al 2008<br>1) Serum potassium < 3 mmol/L AND<br>2) PAC >25 ng/dL AND/OR<br>3) Urinary aldosterone > 30 µg/24 h	152	119	0.316	0.933	0.857	0.516
	Algorithm by Umakoshi et al<br>1) Hypokalemia with serum potassium < 3.5 mmol/L AND<br>2) Unilateral nodule (>1.0 cm) on CT	172	132	0.535	0.811	0.786	0.572
	Algorithm by Umakoshi et al<br>1) Hypokalemia with serum potassium < 3.5 mmol/L AND<br>2) Normal CT OR Unilateral nodule (>1.0 cm) on CT	172	132	0.779	0.424	0.638	0.596
	Algorithm by Kobayashi et al; score of < 8:<br>1) Female sex (1 point)<br>2) PAC < 210 pg/mL (2 points)	151	118	0.848	0.355	0.621	0.652

	3) ARR <620 pg/mL per ng/mL/h (2 points)<br>4) Serum potassium > 3.9 mmol/L (4 points); 3.5-3.9 mmol/L (3 points)<br>5) Absence of adrenal nodules (>1.0 cm) on CT (3 points)						
Umakoshi(2018)	1) Serum potassium < 3.5 mEq/L AND<br>2) CT imaging showing a nodule > 10 mm in diameter	463	1090	0.575	0.898	0.706	0.832
Tamaru(2022)	Machine learning using a random forest (RF) model involving age, sex, body mass index, systolic blood pressure, diastolic blood pressure, PAC, PRA, ARR, albumin, aspartate aminotransferase, alanine aminotransferase, total cholesterol, high-density lipoprotein cholesterol, low-density lipoprotein cholesterol, triglyceride, uric acid, plasma glucose, creatinine, estimated glomerular filtration rate, sodium, potassium, chlorine, and calcium.	33	94	0.697	0.989	0.958	0.903
Kolosova(2022)	Algorithm by Kamemura et al (in development cohort)<br>1) Serum potassium < 3.5 mmol/<br>2) Unilateral nodule ≥ 8 mm<br>3) Baseline ARR ≥ 55<br>4) Male sex	96	54	0.198	0.963	0.905	0.403
	Algorithm by Kobayashi et al (in development cohort)<br>Score of < 8:<br>1) Female sex (1 point)<br>2) PAC < 210 pg/mL (2 points)<br>3) ARR < 620 pg/mL per ng/mL/h (2 points)<br>4) Serum potassium > 3.9 mmol/L (4 points); 3.5-3.9 mmol/L (3 points)<br>5) Absence of adrenal nodules (>1.0 cm) on CT (3 points)	96	54	0.781	0.463	0.721	0.543
**Algorithms involving confirmatory testing alone or combined with biochemical, radiological and demographic characteristics**							
Chen(2021)	PAC post-CCT > 21.2 ng/dL (588 pmol/L)	71	47	0.803	0.766	0.838	0.720
	PAC post-SST > 17.2 ng/dL (477 pmol/L)	71	47	0.887	0.787	0.863	0.822
Kaneko(2019)	PAC post seated SST > 13.1 ng/dL (363 pmol/L)	16	48	0.938	0.792	0.600	0.974
Leung(2019)	Cut off 2 points<br>1) PRA ≤ 0.26 ng/mL/h before saline infusion (1 point)<br>2) PAC ≥ 424 pmol/L after saline infusion (1 point)<br>3) Age at diagnosis < 50 years old (1 point)	38	42	0.842	0.881	0.865	0.860
	Cut off 3 points<br>1) PRA ≤ 0.26 ng/mL/h before saline infusion (1 point)<br>2) PAC ≥ 424 pmol/L after saline infusion (1 point)<br>3) Age at diagnosis < 50 years old (1 point)	38	42	0.316	1.000	1.000	0.618
Minami(2008)	PAC ratio < 1.45 after frusemide upright posture test (2h-post FUP PAC divided by basal PAC)	18	17	0.500	0.938	0.889	0.652

	1) Serum potassium < 3.4 mEq/L AND<br>2) PAC ratio < 1.45 after frusemide upright posture test (2h-post FUP PAC divided by basal PAC)	12	8	0.583	1.000	1.000	0.615
Moriya(2017)	4 positive confirmatory tests – CCT, FUT, SST, AST	17	59	0.882	0.593	0.385	0.946
Nanba(2014)	Score ≥ 5<br>1) Serum potassium ≤ 3.4 mEq/L (2 points)<br>2) PAC ≥ 165 pg/mL (458 pmol/L) (3 points)<br>3) ARR post-CCT ≥ 1000 pg/mL:ng/mL/h (2774 pmol/L:ng/mL/h) (3 points)	32	39	0.750	0.949	0.923	0.822
	Score ≥ 3<br>1) Serum potassium ≤ 3.4 mEq/L (2 points)<br>2) PAC ≥ 165 pg/mL (458 pmol/L) (3 points)<br>3) ARR post-CCT ≥ 1000 pg/mL:ng/mL/h (2774 pmol/L:ng/mL/h) (3 points)	32	39	0.969	0.590	0.660	0.958
	ARR post-CCT ≥ 1067 pg/mL:ng/mL/h (2960 pmol/L:ng/mL/h)	32	39	0.500	0.923	0.842	0.692
Nanba(2015)	SST 2 hour PAC > 282 pg/mL (782 pmol/L)	18	21	0.556	1.000	1.000	0.724
	SST 4 hour PAC > 311 pg/mL (863 pmol/L)	18	21	0.500	1.000	1.000	0.700
	SST 4 hour PAC > 132 pg/ml (366 pmol/L)	18	21	0.778	0.952	0.933	0.833
	SST 2 hour PAC > 119 pg/ml (330 pmol/L)	18	21	0.889	0.810	0.800	0.895
	SST 2 hour PAC > 61 pg/mL (169 pmol/L)	18	21	1.000	NA	NA	NA
	SST 4 hour PAC > 80 pg/mL (222 pmol/L)	18	21	1.000	NA	NA	NA
Puar(2020)	Score of ≥ 1 (Model A) (in development cohort)<br>1) Baseline ARR > 131 ng/dL:ng/mL/h (3634 pmol/L:ng/mL/h) (1 point)<br>2) Post SST PAC > 24 ng/dL (666 pmol/L) (1 point)	70	33	0.678	0.931	0.952	0.587
	Score of ≥ 2 (Model A) (in development cohort)<br>1) Baseline ARR > 131 ng/dL:ng/mL/h (3634 pmol/L:ng/mL/h) (1 point)<br>2) PAC post-SST > 24 ng/dL (666 pmol/L) (1 point)	70	33	0.254	1.000	1.000	0.397
Wada(2021)	Both of the following 60 min after captopril administration:<br>1) ARR > 897 pg/mL/ng/mL/h AND<br>2) PAC > 203 pg/mL (563 pmol/L)	66	172	0.545	0.977	0.900	0.848
	One of the following 60 min after captopril administration:<br>1) ARR > 897 pg/mL/ng/mL/h OR<br>2) PAC > 203 pg/mL (563 pmol/L)	66	172	0.273	0.907	0.529	0.765
Kocjan(2022)	Algorithm by Nagano et al<br>Post-SST PAC > 8.79 ng/dL (244 pmol/L)	59	85	0.898	0.412	0.515	0.854
	Algorithm by Nagano et al<br>PAC reduction rate post-SST < 33.8%	59	85	0.508	0.800	0.638	0.701
Song(2022)	Algorithm by Nanba et al<br>Score of ≥ 3 points (in development cohort)	268	88	0.940	0.227	0.788	0.556

	1) PAC > 16.5 ng/dL (458 pmol/L) (3 points)<br>2) ARR post-CCT ≥ 82 pmol/L:mU/L (3 points)<br>3) Serum potassium K ≤ 3.4 mmol/l (2 point)						
	Algorithm by Nanba et al<br>Score of ≥ 5 points (in development cohort)<br>1) PAC > 16.5 ng/dL (458 pmol/L) (3 points)<br>2) ARR post-CCT ≥ 82 pmol/L:mU/L (3 points)<br>3) Serum potassium K ≤ 3.4 mmol/l (2 point)	268	88	0.880	0.636	0.880	0.636
	Algorithm by Kaneko et al<br>PAC post-SST > 13.1 ng/dL (363 pmol/L) (in development cohort)	268	88	0.851	0.636	0.877	0.583
	Algorithm by Kaneko et al<br>PAC post-SST > 13.1 ng/dL (363 pmol/L) (in validation cohort)	84	117	0.131	0.718	0.250	0.535
Kolosova(2022)	Algorithm by Nanba et al<br>Score > 3 (in development cohort)<br>1) PAC > 16.5 ng/dL (458 pmol/L) (3 points)<br>2) ARR post-CCT ≥ 82 pmol/L:mU/L (3 points)<br>3) Serum potassium K ≤ 3.4 mmol/l (2 point)	96	54	0.396	0.870	0.844	0.448
	Algorithm by Kocjan et al<br>1) Serum potassium < 3.5 mmol/L AND<br>2) PAC post-SST > 18 ng/dL (499 pmol/L) AND<br>3) Unilateral nodule of any size<br>(in development cohort)	96	54	0.281	1.000	1.000	0.439
	Algorithm by Kaneko et al<br>PAC post-SST > 13.1 ng/dL (363 pmol/L) (in development cohort)	96	54	0.677	0.574	0.739	0.500
**Algorithms using biochemical results alone from blood or urine samples**							
Kobayashi(2016)	PAC at 6am > 217.5 pg/mL (603 pmol/L)	32	22	0.900	0.833	0.900	0.833
	PAC at 6:00/PFC at 6:00 with cutoff 17.4 pg/mL:pg/mL	32	22	0.844	0.955	0.964	0.808
	Urinary aldosterone > 14.5 ug/day	29	18	0.759	0.889	0.917	0.696
	PAC > 394 pg/mL (1093 pmol/L)	32	22	0.767	1.000	1.000	0.759
	Urinary aldosterone excretion > 22 ug/day	29	18	0.586	1.000	1.000	0.600
Minami(2008)	Serum potassium < 3.4 mEq/L	18	17	0.833	1.000	1.000	0.850
	Urinary aldosterone ≥ 9 ug/day	14	13	0.786	0.769	0.786	0.769
	PAC ≥ 18 ng/dL (499 pmol/L)	17	16	0.765	0.813	0.813	0.765
Mulatero(2008)	Hypokalaemia </=3.6 mEq/L	31	39	0.742	0.692	0.657	0.771
	Hypokalaemia < 3.0 mEq/L	31	39	0.387	0.949	0.857	0.661
	1) PAC > 25 ng/dL (694 pmol/L) AND	31	39	0.935	0.410	0.558	0.889

	2) Urinary aldo > 30 ug/24h						
Mulatero(2012)	24hr urinary 18OHF > 510 ug/day	20	61	0.300	1.000	1.000	0.813
Nanba(2014)	Serum K ≤ 3.4 mEq/L	32	39	0.688	0.897	0.846	0.778
	PAC ≥ 166 pg/mL (460 pmol/L)	32	39	0.938	0.667	0.698	0.929
Puar(2020)	Aldosterone-to-potassium ratio (APR) > 15 ng/dL:mmol/L (416 pmol/L:mmol/L) (baseline PAC : lowest K ratio) (in development cohort)	70	33	0.457	0.909	0.914	0.441
	APR > 10 ng/dL:mmol/L (277 pmol/L:mmol/L) (in development cohort)	70	33	0.729	0.758	0.864	0.568
	APR > 10 ng/dL:mmol/L (277 pmol/L:mmol/L) (in validation cohort)	48	44	0.521	0.864	0.806	0.623
	Aldosterone to potassium ratio (APR) > 15 ng/dL:mmol/L (416 pmol/L:mmol/L) (baseline PAC : lowest K ratio)	48	44	0.250	0.977	0.923	0.544
Rossi(2012)	PTH > 80 ng/L (8.48 pmol/L)	46	12	0.739	0.833	0.944	0.455
Satoh(2015)	18OHF > 345 ng/dL	113	121	0.540	1.000	1.000	0.699
	18oxoF > 6.1 ng/dL.	113	121	0.301	1.000	1.000	0.605
	18oxoF > 4.7 ng/dL.	113	121	0.832	0.992	0.989	0.863
	PAC > 21.5 ng/dL (597 pmol/L)	113	121	0.814	0.926	0.911	0.842
	18OHF > 234 ng/dL	113	121	0.619	0.959	0.933	0.730
	ARR > 152 ng/dL:ng/mL/h	113	121	0.673	0.901	0.864	0.747
Umakoshi(2018_ 2)	Hypokalaemia < 3.5 mEq/L	482	1109	0.772	0.761	0.584	0.885
Yang(2019)	RF classification trees using peripheral plasma steroid concentrations – in micro-APAs	128	69	0.333	1.000	1.000	0.881
	RF classification trees using peripheral plasma steroid concentrations – in macro-APAs	128	69	0.979	0.804	0.821	0.976
	RF classification trees using peripheral plasma steroid concentrations – in micro- OR macro-APAs	128	69	0.898	0.870	0.927	0.822
Kocjan(2022)	APR > 15	27	32	0.630	0.844	0.773	0.730
Kolosova(2022)	APR > 15 (in development cohort)	96	54	0.500	0.778	0.800	0.467
**Algorithms involving ACTH stimulation or postural stimulation testing**							
Fuss(2022)	Fall in PAC of ≥ 28% after the 4 h postural stimulation test	55	29	0.364	1.000	1.000	0.453
Fuss(2022)	Fall in cortisol of ≥ 10% AND fall in PAC of ≥ 28% after the 4 hour postural stimulation test	35	18	0.514	1.000	1.000	0.514
Jiang(2015)	PAC > 77.90 ng/dL (2161 pmol/L) 120 minutes after ACTH.<br>(1 mg dexamethasone at 12:00 AM, 4 mL (50 IU) IV ACTH at 0800 h.	56	39	0.768	0.872	0.896	0.723

	Blood samples collected at 0, 30, 60, 90, and 120 minutes after injection, and serum cortisol and PAC measured)						
Kaneko(2019)	PAC > 34.2 ng/dL (949 pmol/L) after ACTH stimulation (1 mg dexamethasone at 2300 hours, 0.25 mg of ACTH at 0900 h and PAC measured after 90 minutes of recumbency)	16	48	0.750	0.688	0.444	0.892
Lau(2012)	Fall in PAC ≥ 30% on 1 hr PST	29	14	0.438	0.714	0.778	0.357
	Fall in PAC ≥ 30% on 4 hr PST	29	14	0.556	0.750	0.833	0.429
Moriya(2017)	AUC of PACmax > 605.7 pg/mL (1680 pmol/L) after ACTH stimulation testing	17	59	0.944	0.650	0.447	0.975
	PACmax/cortisol > 18.3 on ACTH stimulation testing (pg/mL:pg/mL)	17	59	0.882	0.831	0.600	0.961
Mulatero(2008)	Increase in PAC ≥ 50% on PST (PAC measured at 0800 h after overnight recumbency and 2 h of standing)	31	39	0.645	0.692	0.625	0.711
	1) Increase in PAC of ≥ 50% on PST (PAC measured at 0800 after overnight recumbency and 2 h of standing) AND<br>2) Abnormal CT scan (with contrast) – nodules or thickening greater than 4 mm	31	39	0.484	0.923	0.833	0.692
Wu(2023)	1) Unilateral lesion on CT (> 10mm) AND<br>2) Negative PST with ≤ 30% increase in PAC (inpatient blood test for PAC and PRA at 0500 after a day in the supine position, a second blood test after 2 hours in the upright position)	314	217	0.344	0.894	0.824	0.485
**Category 5**							
Lau(2012)	CT imaging<br>- Definite nodule with completely normal contralateral gland morphology and adrenal limb thickness not exceeding 5 mm and the body not exceeding 1 cm), OR<br>- Focal nodule with thickened limbs in ipsilateral or contralateral gland	29	14	0.692	0.692	0.818	0.529
Mulatero(2008)	CT scan with contrast – nodules or thickening > 4 mm	31	39	0.871	0.718	0.711	0.875
Umakoshi(2018)	CT imaging	447	1144	0.664	0.688	0.454	0.840
Wu(2023)	Unilateral lesion on CT (> 10 mm)	314	217	0.634	0.613	0.703	0.536

Abbreviations: ACTH: adrenocorticotropic hormone; APR: aldosterone-to-potassium ratio; ARR: aldosterone-to-renin ratio; CT: computed tomography; IU: international unit; IV: intravenous; PA: primary aldosteronism; PAC: plasma aldosterone concentration; PFC: plasma cortisol concentration; PRC: plasma renin concentration; PST: postural stimulation test; SST: saline suppression test.

* Sensitivity, specificity, PPV and NPV were calculated based on applying values reported in publications reviewed to identify the number of cases in the categories of true positive, false positive, true negative and false negative.

Figure S1: QUADAS-2 quality assessment

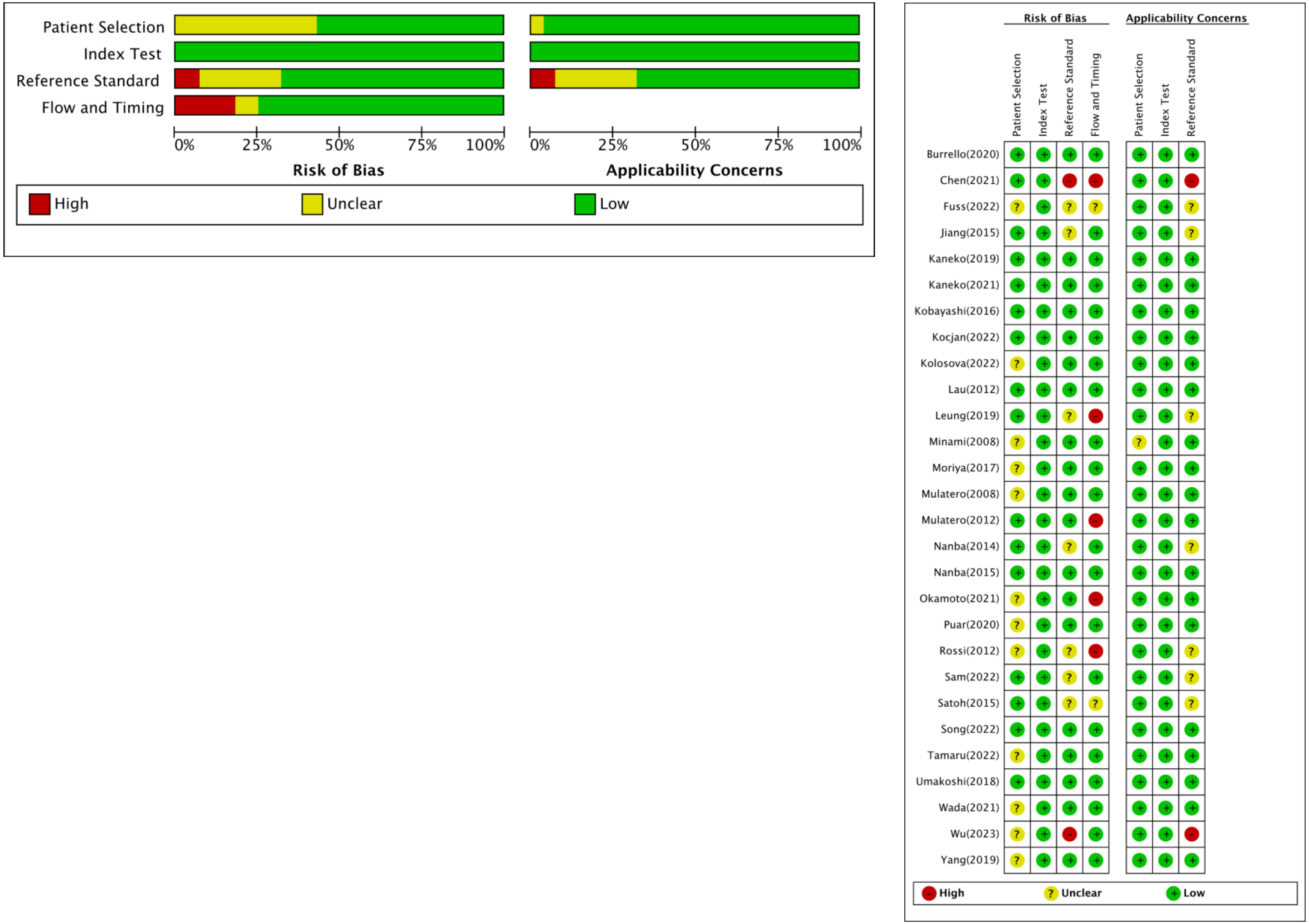

Supplement: Supplementary file 1 — Supporting information. [file CEN-103-3-s001.pdf]
